# Bisphenol A Modifies the Regulation Exerted by Testosterone on 5**α**-Reductase Isozymes in Ventral Prostate of Adult Rats

**DOI:** 10.1155/2013/629235

**Published:** 2013-07-25

**Authors:** Pilar Sánchez, Beatriz Castro, Jesús M. Torres, Asunción Olmo, Raimundo G. del Moral, Esperanza Ortega

**Affiliations:** ^1^Department of Biochemistry and Molecular Biology, Faculty of Medicine, University of Granada, Avenida de Madrid s/n, 18012 Granada, Spain; ^2^Institute of Neurosciences, Faculty of Medicine, University of Granada, 18012 Granada, Spain; ^3^Department of Pathology, San Cecilio University Hospital and School of Medicine, University of Granada, 18012 Granada, Spain

## Abstract

The development, growth, and function of the prostate gland depend on androgen stimulation. The primary androgen in prostate is 5*α*-dihydrotestosterone (DHT) which is synthesized from circulating testosterone (T) through the action of 5*α*-reductase (5*α*-R). Although 5*α*-R occurs as five isozymes, only 5*α*-R1 and 5*α*-R2 are physiologically involved in steroidogenesis. The endocrine disruptor bisphenol A (BPA) alters sexual organs, including the prostate. Our previous findings indicated that BPA decreased the expression of 5*α*-R1 and 5*α*-R2 in rat prostate but also circulating T. Thus, it is unclear whether BPA exerts this effect on 5*α*-R isozymes by reducing circulating T or by any other mechanism. In this study, we examine the effects of short-term exposure to BPA at doses below 25 *μ*g/Kg/d and above 300 *μ*g/Kg/d of the TDI on mRNA levels of 5*α*-R1 and 5*α*-R2 in prostate of adult castrated rats supplemented with T to achieve constant circulating T levels. mRNA levels were measured by absolute quantitative RT-PCR, T levels by RIA, and DHT levels by ELISA. Our results indicated that in castrated rats treated with T BPA at the two doses studied significantly decreased the mRNA levels of both 5*α*-R isozymes in a dose-dependent manner without modifications in circulating T.

## 1. Introduction

The prostate plays a key role in the reproductive mechanism of a man. The prostate is a secretory gland surrounding the urethra at the base of the bladder. As part of the male reproductive system, the prostate gland's primary function is to secrete nutrients, ions, proteins, and hormones that are added to the ejaculatory fluid produced by the seminal vesicles. These molecules are necessary for the survival of the spermatozoids during their journey through the female reproductive tract.

Androgens play a critical role in normal prostatic growth, development, and maintenance throughout the lifespan [1]. Likewise, androgen deprivation at any stage of life causes a decrease in prostate cell number and DNA content [2]. The most potent androgen in the prostate is 5*α*-dihydrotestosterone (DHT) with 10-fold higher potency to induce androgen receptor (AR) signaling than testosterone (T) [3]. DHT is synthesized from circulating T by the enzyme steroid 5*α*-reductase (5*α*-R, EC 1.3.99.5). Of the five types of 5*α*-R enzymes characterized to date, only type 1 (5*α*-R1) and type 2 (5*α*-R2) are believed to be physiologically involved in steroidogenesis [4]. 5*α*-R1 isozyme occurs in tissues such as the liver [5], the skin, and the brain, where it acts as a key enzyme in the biosynthesis of neuroactive steroids [6–9], although it is also expressed in the prostate [10, 11]. 5*α*-R2 isozyme is predominantly expressed in male reproductive tissues including the prostate [10–13]. Normal growth, development, and function of the prostate are mainly regulated by 5*α*-R2, with 5*α*-R1 also contributing to these events [10, 14, 15].

A wide range of environmental and industrial chemicals can disrupt growth, development, and normal function of the prostate by interfering with the physiological action of both androgens and estrogens. Bisphenol A (BPA) is an environmental endocrine disruptor which has been shown to mimic the estrogenic action [16] and may also modify the androgenic action [17, 18]. BPA is used extensively in the production of polycarbonate plastics, epoxy linings of food and beverage cans, and in dental products [19, 20]. Exposure to BPA is nearly universal. In a recent study, BPA was detected in urine samples from 92.6% of the US population examined [21].

Our research group has recently demonstrated that the short-term administration of BPA to adult rats is associated with a decrease in the expression of both 5*α*-R1 and 5*α*-R2 isozymes [22]. The change in 5*α*-R isozymes levels may cause subsequent prostate dysfunction by interfering with androgens metabolism. We also found decreased plasma T levels in BPA-treated rats. Our previous study showed that 5*α*-R1 and 5*α*-R2 are positively regulated by T within the prostate [10]. Therefore BPA may exert its downregulating effects on 5*α*-R isozymes by reducing T levels or by other mechanisms.

In order to shed light on this question, in this study we have examined the effects of short-term BPA exposure on the expression of 5*α*-R1 and 5*α*-R2 isozymes in ventral prostate of adult castrated rats supplemented with constant doses of T.

## 2. Materials and Methods

### 2.1. Animals and Treatments

Experiments were performed strictly in accordance with recommendations in the Guide for the Care and Use of Laboratory Animals of the National Institutes of Health. Animal care and experimental procedures were approved by the Animal Experimentation Ethics Committee of the University of Granada, Spain (Ref. 412-2012).

Adult male Wistar rats weighing 260–280 g were housed in an air-conditioned room with fluorescent lights on from 08:00 to 20:00 and given standard laboratory pellet chow (Panlab rodent chow, Barcelona, Spain). Although the concentration of phytoestrogens in the diet was not evaluated, all animals were exposed to the same phytoestrogen levels because the food intake was equivalent for BPA-treated and nontreated rats. Exposure to environmental endocrine disruptors was minimized by housing the rats in stainless steel cages and using glass bottles with rubber stoppers to supply them with tap water. The experimental groups studied were castrated rats (C), castrated rats plus T (C + T), castrated rats plus T plus BPA (25 *μ*g/Kg/d) (C + T + BPA25), and castrated rats plus T plus BPA (300 *μ*g/Kg/d) (C + T + BPA300). All animals underwent bilateral orchidectomy under ether anesthesia. A week later, with the exception of group C, all rats were subcutaneously (s.c.) daily injected with 500 *μ*g of testosterone propionate (Sigma-Aldrich, St. Louis, MO, USA) dissolved in sesame oil vehicle for four days. 30 min before the administration of T, the rats grouped in C + T + BPA25 and C + T + BPA300 were s.c. daily injected with BPA (Sigma-Aldrich, St. Louis, MO, USA) dissolved in sesame oil vehicle at doses of 25 *μ*g/Kg/d and 300 *μ*g/Kg/d, respectively, for four days. Group C was s.c. daily injected with oil vehicle alone. We have followed the protocol of Leranth et al. [23], but we have also administered BPA at dose of 25 *μ*g/Kg/d in order to study the effects of BPA at doses under the Tolerable Daily Intake (50 *μ*g/Kg/d). 30 min after the last injection of T, rats were euthanized by decapitation, and the prostate was removed, weighed, frozen in liquid nitrogen, and stored at −80°C until analysis. Blood samples were collected in heparinized tubes. The blood was centrifuged at 2000 rpm for 10 min. The plasma was separated and stored at −20°C until the hormone analysis. Each study group comprised 8 animals.

### 2.2. Hormone Assays

Plasma T concentrations were measured by RIA using a commercial (DiaSorin Vercelli, Italy) kit without modifications; intra- and interassay coefficients of variation were 7.6% and 12.0%, respectively, and the sensitivity was 0.05 ng/mL. Plasma DHT concentrations were measured by direct ELISA using a commercial (Diagnostics Biochem Canada, Inc., Ontario, Canada) kit; intra- and inter-assay coefficients of variation were 5.9% and 7.5%, respectively, and the sensitivity was 6.0 pg/mL. 

### 2.3. RNA Extraction and Reverse Transcription

Total RNA was extracted from 50 mg of rat ventral prostate tissue with Trizol reagent (Invitrogen) according to the instructions of the* Sanger Institute*. RNA samples were then treated with Turbo-DNAse (Ambion) to remove any contamination with genomic DNA. The quantity and purity were determined by using a NanoDrop ND-1000 spectrophotometer (A260/280 ratio), and the integrity was tested by means of denaturing gel electrophoresis. First-strand cDNA was synthesized from 1 *μ*g of total RNA by using MuLV reverse transcriptase (Applied Biosystems). The following agents were added to a final volume of 20 *μ*L reaction: 5 mM MgCl_2_, 1 × RT buffer, 1 mM each dNTP, 1 U/*μ*L RNase inhibitor, 2.5 U/*μ*L MuLV reverse transcriptase, 2.5 *μ*M Oligo (dT)_16_, and 1 *μ*g total RNA. Reactions were incubated at 42°C for 15 min, followed by 5 min at 99°C. 

### 2.4. Quantitative Real-Time PCR

Absolute quantification of mRNA levels of 5*α*-R1 and 5*α*-R2 in rat prostate tissues was performed by real-time PCR using the Techne Quantica real-time PCR system with SYBR Green PCR Master Mix (Promega). In comparison to relative quantification, this method offers the advantage of giving an absolute copy number for a specific target. The amount of mRNA was expressed as the number of mRNA copies per micrograms of total RNA. We amplified tissue samples by real-time PCR in parallel with competitive cRNA standard curves generated following the method described by Fronhoffs et al. [24].

The PCR profile was as follows: denaturation at 94°C for 30 seconds, annealing at 55°C for 30 seconds, and extension at 72°C for 30 seconds. The number of cycles was 40 in all cases. At the end of the amplification phase, a melting curve analysis was carried out on the products formed in order to confirm that a single PCR product was detected by the SYBR Green dye. 

Primers for 5*α*-R1 (*Srd5a1* mRNA, Genbank accession n° NM_017070.3) and 5*α*-R2 (*Srd5a2* mRNA, Genbank accession n° NM_022711.4) were designed using Primer 3 software. The primer sequences (5′-3′) are given in Table 1.

### 2.5. Statistical Analysis

Statistical analysis of the results was performed using the Student's *t*-test. Data are expressed as mean ± SD. The statistical analysis was performed using the STATA Version 10 (Stata Corp. 2007) software.

## 3. Results

### 3.1. Plasma T and DHT Levels

**Figure 1:**
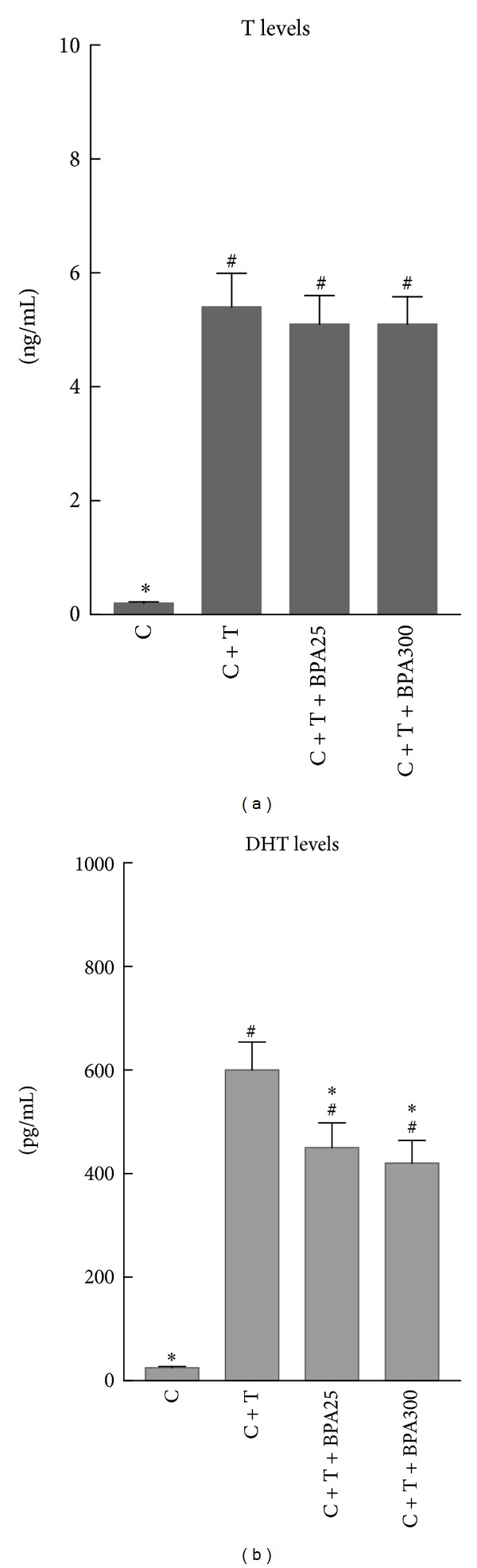
Plasma testosterone (T) levels (a) and plasma dihydrotestosterone (DHT) levels (b) in castrated rats (C), castrated rats supplemented with T (C + T), and castrated rats supplemented with T plus BPA at doses of 25 *μ*g/Kg/d (C + T + BPA25) and 300 *μ*g/Kg/d (C + T + BPA300) for 4 days. ^#^ *P* < 0.05 or less versus C group. *P < 0.05 or less versus C + T group.

Castrated animals had lower T levels. All groups treated with T had a significant increase in plasma T levels in comparison with castrated rats without T treatment (Figure 1(a)). Both groups treated with BPA at the doses of 25 *μ*g/Kg/d and 300 *μ*g/Kg/d had similar plasma T levels, with no significant differences in comparison with T-treated castrated rats (Figure 1(a)). Castrated animals had lower DHT levels. All groups treated with T had a significant increase in plasma DHT levels in comparison with castrated rats without T treatment (Figure 1(b)). Both groups treated with BPA at the doses of 25 *μ*g/Kg/d and 300 *μ*g/Kg/d had lower plasma DHT levels, in comparison with T-treated castrated rats, with no significant differences between both BPA-treated groups (Figure 1(b))

### 3.2. 5*α*-R1 and 5*α*-R2 mRNA Levels

**Figure 2:**
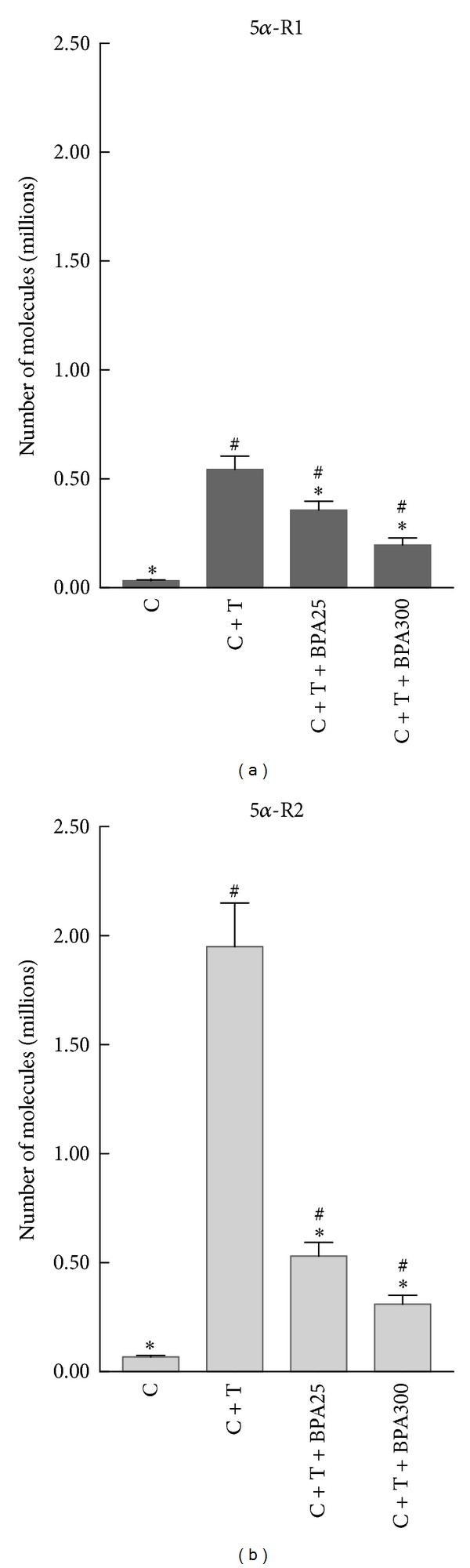
mRNA levels of 5*α*-R1 (a) and 5*α*-R2 (b) in prostate of castrated rats (C), castrated rats supplemented with T (C + T), and castrated rats supplemented with T plus BPA at doses of 25 *μ*g/Kg/d (C + T + BPA25) and 300 *μ*g/Kg/d (C + T + BPA300) for 4 days. ^#^ *P* < 0.05 or less versus C group. *P < 0.05 or less versus C + T group.

All groups treated with T had a significant increase in 5*α*-R1 mRNA levels in comparison with castrated rats without T treatment (Figure 2(a)). Both groups treated with BPA at the doses of 25 *μ*g/Kg/d and 300 *μ*g/Kg/d had significant decreased 5*α*-R1 mRNA levels in comparison with castrated rats treated with T (Figure 2(a)). In addition, BPA-treated rats at dose of 300 *μ*g/Kg/d had significant decreased 5*α*-R1 mRNA levels when compared with BPA-treated rats at dose of 25 *μ*g/Kg/d (Figure 2(a)).

All groups treated with T had a significant increase in 5*α*-R2 mRNA levels in comparison with castrated rats without T treatment (Figure 2(b)). Both groups treated with BPA at the doses of 25 *μ*g/Kg/d and 300 *μ*g/Kg/d had significant decreased 5*α*-R2 mRNA levels in comparison with castrated rats treated with T (Figure 2(b)). In addition, BPA-treated rats at dose of 300 *μ*g/Kg/d had significant decreased 5*α*-R2 mRNA levels when compared with BPA-treated rats at dose of 25 *μ*g/Kg/d (Figure 2(b)).

BPA produced a much greater reduction on 5*α*-R2 (*P* < 0.01) than that observed for 5*α*-R1 (*P* < 0.05) mRNA levels.

### 3.3. Prostate Weight

**Figure 3:**
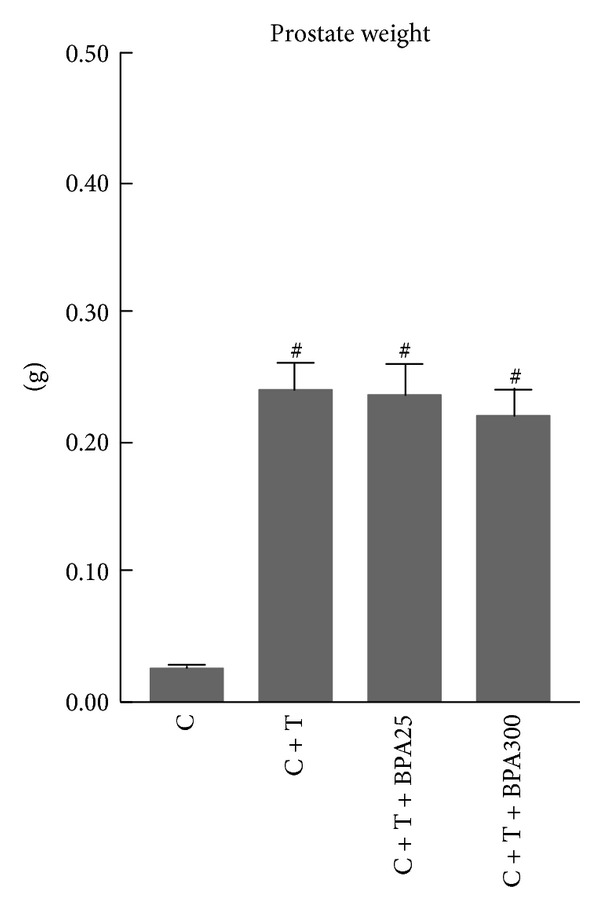
Prostate weight of castrated rats (C), castrated rats supplemented with T (C + T), and castrated rats supplemented with T plus BPA at doses of 25 *μ*g/Kg/d (C + T + BPA25) and 300 *μ*g/Kg/d (C + T + BPA300) for 4 days. ^#^ *P* < 0.05 or less versus C group.

All groups treated with T had a significant increase in prostate weight in comparison with castrated rats without T treatment (Figure 3). Both groups treated with BPA at the doses of 25 *μ*g/Kg/d and 300 *μ*g/Kg/d had similar prostate weight, with no significant differences in comparison with T-treated castrated rats (Figure 3).

## 4. Discussion

The normal development and function of the adult prostate are dependent on the action of DHT, which is synthesized within the prostate from circulating T by the enzyme 5*α*-R. Of the five types of 5*α*-R enzymes characterized to date, only 5*α*-R1 and 5*α*-R2 are believed to be physiologically involved in steroidogenesis [25]. 5*α*-R1 and 5*α*-R2 are mainly expressed in epithelial and stromal cells, respectively [26]. In epithelial cells, 5*α*-R1 isozyme may be responsible for synthesizing DHT, which acts in an autocrine manner to stimulate their differentiation. Alternatively, DHT may act in paracrine fashion to stabilize or stimulate the division of the adjacent androgen-dependent luminal epithelium [26]. Expression of 5*α*-R2 isozyme in the stroma of the prostate is consistent with the central role played by these cells in the development of the gland [26]. Given the important role that both isozymes play in the prostate function, several factors are responsible for the *Srd5a1* and *Srd5a2* gene regulation, including T as we previously demonstrated [10]. 

The results of the present study show that the administration of T for four days exerts an upregulation of both 5*α*-R1 and 5*α*-R2 isozymes, with this effect being greater for 5*α*-R2. Our group previously reported that both 5*α*-R isozymes were positively regulated by T [10]. However, in this study we have used a different T dose, a different injection schedule, and a more accurate technology to quantify the mRNA levels. We previously quantified 5*α*-R isozymes mRNA levels by competitive RT-PCR coupled with laser-induced fluorescence capillary electrophoresis (LIF-CE) using a mimic DNA as internal standard [27]. However, this method appears to have some drawbacks which may impair the efficiency of PCR amplification, that is, different size and nucleotide sequence between target cDNA and competitive standard DNA and the fact that cDNA obtained from RT reaction is a mixed RNA/DNA molecule while competitive standard is added in DNA form. We have now circumvented these limitations by using another approach for the accurate measurement of gene transcript levels, which is based on real-time PCR in combination with a rapid and simple method for the construction of simultaneously amplified cRNA standard curves with identical size and nucleotide sequences as the target gene [24].

According to the present results, short-term BPA administration to castrated rats supplemented with T decreased the mRNA levels of both 5*α*-R1 and 5*α*-R2 isozymes in ventral prostate of rats, which had constant levels of circulating T. BPA has been shown to exert endocrine-disrupting effects on reproduction, development, and metabolism [28]. Recent findings linked exposure to BPA with several male reproductive disorders [29–32] and prostate diseases [33, 34]. Our group recently demonstrated that adult exposure to BPA decreased in the rat prostate gland the expression of both 5*α*-R1 and 5*α*-R2, as well as the circulating T levels [22]. The latter finding is not surprising to us since BPA decreases luteinizing hormone (LH) secretion [35] and inhibits T biosynthetic enzymes in the testis [36]. Because within the prostate 5*α*-R1 and 5*α*-R2 are positively regulated by T [10], BPA may downregulate both 5*α*-R isozymes by decreasing T levels, but in our study the circulating levels of T were constant. A possible explanation to this fact may be the circulating levels of DHT which are decreased in our BPA-treated rats. In this context, the effects of BPA on other tissues where 5*α*-reduction occurs such as liver and skin should be also kept in mind to explain the diminished circulating DHT levels. Our previous finding showed that DHT exerts a positive effect on the genetic expression of both 5*α*-R isozymes in the prostate, although with a lesser extent than that observed for T [10]. In vitro studies reveal that when DHT formation is inhibited it will induce a pronounced downregulation of androgen receptor (AR) mRNA levels [37], which may lead to the downregulation of 5*α*-R isozymes [22].

Another intriguing possibility is that BPA may directly affect AR function via multiple mechanisms: (1) antagonizing AR signaling, (2) avoiding AR translocation to the nucleus, (3) avoiding AR interaction with its coactivator and its subsequent transactivation [17, 18], and (4) decreasing the number of AR in stromal cells of the rat ventral prostate [38]. As have been indicated by Teng et al. [17], BPA binds to AR and competes with androgen binding at the LBD region of the receptor. Like known androgen antagonists, BPA is unable to promote the formation of functional AR foci in the nucleus. Thus, BPA binding to AR interferes with nuclear receptor translocation.

In this study, BPA treatment did not alter ventral prostate weight, despite the down-regulation of both 5*α*-R isozymes and presumably lower intraprostatic DHT levels, in view of circulating DHT levels. Two points may explain this fact: other additional factors are contributing to the growth of the prostate, and/or the BPA regime followed in this study is not appropriate to induce observable changes in prostate weight. Other authors have shown that prenatal exposure to low dose of BPA increases prostate size [38–40] and these changes persisted during adulthood. The effects of BPA on prostate weight could be dose dependent [39], but also they could be dependent of the BPA administration schedule and the life stage when the exposure occurs. 

## 5. Conclusion

Our results showed that BPA decreases the positive regulatory effect that T exerts on 5*α*-R1 and 5*α*-R2 gene regulation in the prostate, at least in part, independently of circulating T. Given the important role of 5*α*-R isozymes in the physiological function of the prostate, the exposure to BPA should be considered harmful for this gland.

## Figures and Tables

**Table 1:** Primer sequences (5′-3′) for PCR amplification.

Primers	Forward primer	Reverse primer
5*α*-R1	GAGATATTCAGCTGAGACCC	TTAGTATGTGGGCAGCTTGG
5*α*-R2	ATTTGTGTGGCAGAGAGAGG	TTGATTGACTGCCTGGATGG
